# Decision-making under explicit risk is impaired in multiple sclerosis: relationships with ventricular width and disease disability

**DOI:** 10.1186/s12883-015-0318-0

**Published:** 2015-04-23

**Authors:** Ashley D Radomski, Christopher Power, Scot E Purdon, Derek J Emery, Gregg Blevins, Kenneth G Warren, Esther Fujiwara

**Affiliations:** Department of Psychiatry, University of Alberta, 1E1.01 WCM Health Sciences Centre, Edmonton, Alberta T6G 2R7 Canada; Department of Medicine (Neurology), University of Alberta, Edmonton, Canada; Neuropsychology Service, Alberta Hospital Edmonton, Edmonton, Canada; Department of Radiology and Diagnostic Imaging, University of Alberta, Edmonton, Canada

**Keywords:** Multiple sclerosis, Cognition, Decision-making, Neuropsychological tests, MRI, Central atrophy

## Abstract

**Background:**

Decision-making is an essential function of everyday life. Decision-making under explicit risk requires developing advantageous decision strategies based on fixed outcomes (e.g., probabilities of winning or losing a bet). Decision-making and its neural substrates have been rarely studied in MS. We expected performance in decision-making under risk to be lowered in MS patients, and negatively correlated with disease-related disability, cognition, and ventricular width.

**Methods:**

Three groups were included: 32 MS patients and 20 healthy controls were examined with conventional neuropsychological tests and the Game-of-Dice Task (GDT) assessing decision-making under explicit risk. Linear 2-D ventricular width was assessed on MS patients’ clinical MRIs and compared to a third group, 20 non-MS neurological control patients.

**Results:**

Compared to healthy controls, MS patients showed impaired GDT and neuropsychological performance, depending on the MS-subtype (relapsing-remitting (RR), n = 22; secondary progressive, n = 10) and disability severity among RR-MS patients. In MS patients, GDT performance correlated with processing speed, intercaudate ratio, and third ventricle ratio (p’s < 0.05). Mediation analysis showed that the link between GDT performance and processing speed was fully explained by ventricular size.

**Conclusion:**

Decision-making under explicit risk was reduced in MS patients, but only those with more pronounced disability. Independent of processing speed, decision-making under explicit risk correlates inversely with central atrophy in MS.

**Electronic supplementary material:**

The online version of this article (doi:10.1186/s12883-015-0318-0) contains supplementary material, which is available to authorized users.

## Background

Multiple sclerosis (MS) is a demyelinating, inflammatory disease of the central nervous system involving both white matter and grey matter changes [1]. Cognitive impairment, especially in processing speed and attention [2,3], occurs in 40-60% of patients [3], and has been linked to neurodegenerative changes [4,5]. Manual 2-D measures of cranial ventricular width on magnetic resonance images (MRI) are a simple way to estimate loss of neighbouring brain parenchyma. These measures have been used for many decades, have shown satisfactory agreement with high-resolution 3-D MRI in MS, and have been associated with disease severity, progression, and cognitive status in MS patients [2,6-13]. The third ventricle width/ratio and intercaudate distance/intercaudate ratio, often referred to as reflecting ‘central atrophy’, seem particularly sensitive to cognitive dysfunction in MS [2,6,14,15].

Decision-making is a complex function with high relevance to everyday life. Decision-making requires choosing between options and using feedback from prior choices to develop and maintain an optimal choice strategy. The Game-of-Dice Task (GDT [16]) assesses decision-making under explicit risk and emphasizes the cognitive aspects of decision-making by providing information about winning/ losing probabilities associated with each choice. In contrast to decision-making under explicit risk, in decision-making under *ambiguity* the odds for each choice option are not made explicit. Instead, the goal is to implicitly learn choice-outcome contingencies solely by feedback and trial-and-error. The most prominent task assessing decision-making under ambiguity is the Iowa Gambling Task (IGT [17]). A number of studies using the IGT [17] have found impairments in MS, but these were usually independent of cognitive deficits [18-22]. Two previous MS studies used the GDT. Farez and others (2014) [23] reported deficits in 27 relapsing-remitting MS patients with minimal disability (mean Expanded Disability Status Scale [EDSS] [24] = 1.03) and short disease duration (mean = 7.9 months). Patients’ GDT performance was related to processing speed and visual memory performance [23]. Conversely, Cogo and others (2014) [25] found 60 relapsing-remitting MS patients with minimal disability (mean EDSS = 1.4) but longer disease duration (mean = 40.8 months) unimpaired on the GDT. In their study, GDT performance in MS patients was unrelated to other cognitive functions. These findings require further clarification.

Patients with several primary neurodegenerative disorders have shown deficits on the GDT and other decision-making tasks [26]. In MS, one decision-making study (using the IGT) [18] tested patients with a relatively long disease duration (median = 8.58 years) and higher levels of disability (median EDSS = 2.0), i.e., individuals with probable neurodegenerative changes due to the progression of MS. In this study, decision-making deficits on the IGT were observed only in MS patients with EDSS > 2. However, direct neuroanatomical substrates of decision-making in MS are rare. Roca et al. [20] tested decision-making in the IGT in 12 MS patients in conjunction with diffusion tensor imaging (fractional anisotropy and apparent diffusion coefficient) along frontal lobe white matter bundles (orbito-frontal, fronto-lateral, fronto-medial and gyrus cinguli regions). They reported no association between the structural integrity along any of these frontal white matter bundles and decision-making performance in their MS patients. A recent larger-scale study [27] with 105 MS patients with varying levels of disability examined another decision-making paradigm, the Cambridge Gambling Task (CGT). The authors used the CGT in combination with diffusion MRI (diffusion orientational complexity), measurement of grey matter volumes, and white matter lesion volume. The decision-making speed in the CGT in particular was impaired in the MS group, especially in secondary progressive MS patients (n = 26). Decision-making speed in the MS group was correlated with cognitive functions (processing speed, memory, executive functions), and with diffusion orientational complexity (grey matter pathology) in the medial prefrontal, middle frontal gyrus, anterior cingulate and the caudate, as well as with white matter lesion volumes. This network of medial frontal-caudate regions matches well with previous decision-making findings from primary neurodegenerative disorders [26]. However, it should also be noted that the CGT is a speeded task, unlike the GDT. Muhlert et al. [27] did not report whether the identified correlations between grey/white matter pathologies and decision speed were also present for processing speed in general. Of note, another study using the CGT [28] also found decision speed in the CGT correlated with processing speed in MS. Thus, using the CGT as a measure, it remains possible that slowing in processing speed also contributes to problems with speeded decision-making in MS and therefore may share neural substrates.

Therefore, our goals with the current study were to test whether the GDT as a non-speeded measure of decision-making under explicit risk is impaired in MS, and to evaluate whether GDT performance is linked to increasing disability (defined here as functional impairment according to the EDSS [24]) and to atrophic brain changes. Using measures of ventricular width to approximate brain atrophy, we included MS patients with EDSS scores between 0 and 6.5. The following hypotheses were tested: We expected that GDT performance would be impaired in MS as a function of disability and ventricular width. Because the GDT is usually correlated with executive functions in non-MS populations [16,29,30], we expected to observe a correlation between the GDT and executive function. However, one MS study with the GDT [23] found correlations between decision-making and processing speed. Therefore, we tested whether our group would also show correlations between GDT performance and processing speed. Finally, we tested whether potential associations between GDT performance and ventricular width were mediated by other cognitive functions.

## Methods

This study was conducted in adherence to the Declaration of Helsinki and with approval from the University of Alberta Health Research Ethics Board (ethics file numbers: Pro00007274; Pro00041844). All participants provided written informed consent.

### Participants

The MS patients (n = 32) were recruited through the Northern Alberta Multiple Sclerosis Clinic in Edmonton. Healthy controls (n = 20) were recruited through online and print advertisements. Exclusion criteria for all participants were: a) present or past major neurological or psychiatric condition(s), apart from MS for the patients, b) substance abuse within the past 5 years, c) uncorrected vision/hearing problems, d) non-fluency in English, e) current corticosteroid treatment. Only patients with a diagnosis of relapsing-remitting (RR-) or secondary progressive (SP-) MS (revised McDonald criteria [31]) and an EDSS [24] score < 7 were included.

Due to our sampling and inclusion of a large range of EDSS scores, the variability of disability within the RR subtype was rather high. In order to explore potential decision-making differences within the heterogeneous group of RR-MS patients and to equate sample sizes, patients were further sub-divided into three groups based on disability and subtype: (1) RR-1: RR-MS patients with EDSS < 3.0, indicating no to minimal disability (n = 13), (2) RR-2: RR-MS patients with EDSS ≥ 3.0, indicating moderate disability (n = 9), and (3) SP: SP-MS patients with EDSS ≥ 3.0 (n = 10). The rationale for further splitting of the RR-MS subgroup was to illustrate a gradient of possible decision-making (and other cognitive) deficits as a function of disability within the larger group of patients with the RR subtype. Our choice of EDSS cut-off score was motivated such that in the EDSS, a score less than 3 indicates at most, mild disability in one functional system or minimal disability in two functional systems. Scores 3 and higher include moderate disability levels, which we intended to distinguish from minimal-mild disability levels.

MS patients were currently taking a variety of prescription medications for health conditions not part of the exclusion criteria, including high blood pressure, high cholesterol, heartburn and acid reflux, osteoarthritis and bone density, asthma, and gastrointestinal/urological concerns. Participants on the antidepressants bupropion, selective serotonin reuptake inhibitors and selective norepinephrine reuptake inhibitors were permitted. Since it is common for MS patients (especially SP-MS patients) to be prescribed psychotropic drugs for symptom management (i.e. insomnia, neuropathic pain), these patients were not excluded from the study, even though these medications have the potential to impact cognition. Thus, some MS patients were on low doses of benzodiazepines and anticonvulsants. Current self-reported medications were obtained from all 32 MS patients. In total, 9 RR-MS patients (28.13%, 5 RR-1, 4 RR-2) were currently prescribed a disease-modifying drug (i.e., interferon beta or glatiramer acetate), with no patients prescribed both interferon beta and glatiramer acetate simultaneously. No SP-MS patients were prescribed disease-modifying drugs. Twenty-one patients (65.63%; 8 RR-1, 6 RR-2, 7 SP) were taking at least one prescription medication for MS and/or neurological concerns. Ten patients (31.25%; 4 RR-1, 1 RR-2, 5 SP) were on more than one medication. In total, two patients (6.25%) were on a medication for neuropathic pain (1 RR-2, 1 SP), five patients (15.63%) were prescribed a medication for sleep and/or anxiety (2 RR-1, 3 SP), twelve patients (34.38%) were prescribed antidepressants (6 RR-1, 1 RR-2, 4 SP), and nine patients (28.13%) were on anti-spasticity or muscle relaxation medications (2 RR-1, 2 RR-2, 5 SP).

Table 1 shows healthy controls were comparable to MS patients in gender distribution, age, education, and estimated premorbid IQ [32]. Disease duration and age at onset of MS were statistically not different among patient subgroups, although it should be noted that the RR-2 group had on average a 6–7 years shorter disease duration than both the RR-1 and the SP subgroup. This pattern reflects that the RR-1 subgroup would have had relatively little functional impairment over long periods of time, sometimes called ‘benign MS’. The exact criteria and existence of such a subtype are widely debated, therefore we retain the more neutral RR-1 label here [33-36]. In agreement with an interpretation of a more benign disease course in the RR-1 group, the Multiple Sclerosis Severity Score (MSSS; [37]) indicated a more aggressive disease course in the RR-2 and SP groups than in RR-1.

**Table 1:** **Background variables**

	**HCs (n = 20)**	**MS (n = 32)**	**Test**	**RR-1 (n = 13)**	**RR-2 (n = 9)**	**SP (n = 10)**	**Test**
**Female, n (%)**	12 (60%)	24 (75%)	χ^2^ = 1.3	11 (84.6%)	6 (66.7%)	7 (70%)	χ^2^ = 2.28
			p = 0.47				p = 0.60
**Age, years**	48.2 (11.0)	50.81 (9.5)	t = 0.9	52.0 (9.8)	46.8 (10.7)	52.9 (7.5)	F = 0.96
			p = 0.37				p = 0.55
**Education, years**	14.7 (2.1)	13.6 (1.7)	t = −2.0	14.2 (2.1)	13.2 (1.7)	13.4 (0.9)	F = 1.88
			p = 0.051				p = 0.15
**Premorbid IQ**	110.2 (11.7)	105.2 (12.2)	t = −1.48	110.9 (10.3)	101.2 (15.3)	101.4 (9.0)	F = 2.53
			p = 0.15				p = 0.07
**MS-onset age, years**	--	34.4 (9.7)	--	34.1 (9.2)	35.1 (9.7)	34.3 (11.2)	F = 0.03
							p = 0.98
**Disease duration, years**	--	15.9 (10.3)	--	17.2 (12.8)	11.3 (7.5)	18.4 (8.4)	F = 1.32
							p = 0.28
**EDSS score (medians, ranges)**	--	3 (0–6.5)	--	2 (0–2.5)	3.5 (3–6.5)	6 (3–6.5)	--
**MSSS**	--	3.9 (2.8)	--	1.7 (2.0) ^a^	5.5 (2.5) ^b^	5.3 (1.8) ^b^	F = 12.0
							**p < 0.001**
**Disease-modifying therapy** ^**1**^	--	9 (28.1%)	--	5 (38.5%)	4 (44.4%)	0 (0.0%)	--

*Abbreviations:* *EDSS* Expanded Disability Status Scale; *HCs* Healthy controls; *MSSS* Multiple Sclerosis Severity Score; *RR-1* Relapsing-remitting MS patients with EDSS scores 0–2.5; *RR-2* Relapsing-remitting MS patients with EDSS scores ≥ 3; *SP* Secondary progressive MS patients.

^1^ Interferon beta 1a or 1b or glatiramer acetate.

^ab^ Different superscripts indicate significant between-group differences in post-hoc t-tests, Bonferroni-corrected.

Data are means (standard deviations) unless stated otherwise.

In order to provide a reference for the ventricular width measures we applied to retrospectively collected cranial MRIs of the MS patients (see section *MRI scans*), we included a third group. This group were 20 patients who had undergone cranial MRI scanning for the following reasons: Syncope: n = 1, cranial nerve palsy: n = 1, transverse myelitis: n = 2, encephalitis: n = 2, systemic lupus erythematosus: n = 2, optic neuritis: n = 1, lacunar stroke: n = 1, systemic lupus erythematosus cerebritis: n = 1, epilepsy: n = 1, seizure: n = 3, brainstem stroke: n = 2, spinal cord stroke: n = 1, cerebelitis, transient ischemic attack: n = 1, Sneddon’s Syndrome: n = 1).

**Table 2:** **Cognitive composites (z-scaled on study HCs) and GDT performance**

**Neuropsychological domain/test**	**HCs (n = 20)**	**All MS patients (n = 32)**	**Test**	**RR-1 (n = 13)**	**RR-2 (n = 9)**	**SP (n = 10)**	**Test**
**Processing speed composite score**	0.00 (0.76)	−0.81 (0.81)	t = 3.58	−0.45 (0.60)	−1.04^*^ (0.61)	−1.07^*^ (1.07)	F = 6.13
			**p = 0.001**				**p = 0.001**
**Memory composite score**	0.00 (0.93)	−1.54 (1.16)	t = 5.02	−1.19^*^ (1.23)	−1.57^*^ (1.36)	−1.97^*^ (0.76)	F = 9.59
			**p < 0.001**				**p < 0.001**
**Executive functions composite score**	0.00 (0.59)	−0.63 (0.69)	t = 3.41	−0.53 (0.85)	−0.85^*^ (0.71)	−0.58 (0.39)	F = 4.30
			**p = 0.001**				**p = 0.009**
**Global cognitive functions composite score**	0.00 (0.63)	−0.93 (0.63)	t = 5.12	−0.61^*^ (0.65)	−1.11^*^ (0.71)	−1.18^*^ (0.35)	F = 10.54
			**p < 0.001**				**p < 0.001**
**GDT – net-score (raw score)**	12.5 (7.6)	6.8 (10.9)	t = 2.1	13.69 (5.82)	0.22^*^ (11.77)	3.80^*^ (10.73)	F = 4.87
			**p = 0.046**				**p = 0.01**
**GDT – strategy shifts (raw score; medians/ranges)**	1 (0–10)	3.5 (0–12)	U = 1.6	2 (0–8)	4 (0–10)	5.5^*^ (1–12)	χ^2^ = 41.0
			p = 0.069				**p = 0.009**

*Abbreviations:* *HCs*: Healthy controls; *GDT* Game-of-Dice Task; *RR-1* Relapsing-remitting MS-patients with EDSS (Expanded Disability Status Scale) scores 0–2.5; *RR-2* Relapsing-remitting MS-patients with EDSS scores ≥ 3; *SP* Secondary progressive MS-patients.

^*^Significant difference to healthy controls (post-hoc Dunnett t-test or Bonferroni-corrected U-test).

Data are means (standard deviations) or medians (ranges).

### Neuropsychological tests

The neuropsychological test battery included conventional tests of processing speed (Symbol-Digit Modality Test, Paced Auditory Serial Addition Test – 3-second version, 9-hole Pegboard, Forward Digit Span), verbal memory (Verbal Selective Reminding Task, SRT), and executive functions (intrusions in the SRT, Wisconsin Card Sorting Test, Phonemic Fluency, Tower of Hanoi, Backward Digit Span). Acknowledging that neuropsychological measures, especially executive function tests, are not process-pure and are combinable in multiple ways, we attempted to minimize type-I error by reducing the number of comparisons. Therefore, we summarized the individual test scores into three composite z-scores based on the performance of the healthy controls in our study: processing speed, memory, and executive functions. A global cognitive function z-score was derived by averaging the three composite scores (see Table 2). Psychosocial and symptom questionnaires were administered to MS patients only (Hospital Anxiety and Depression Scale, Fatigue Assessment Inventory, Dysexecutive Questionnaire, London Handicap Scale). Test references, assignment of individual tests to the composite scores, and individual test results are shown in Table 3. Additional file 1 lists references and norm-based results of the MS patients in the questionnaires.

**Table 3:** **Performance in individual neuropsychological tests**

**Neuropsychological domain/test**	**HCs (n = 20)**	**All MS patients (n = 32)**	**Test**	**RR-1 (n = 13)**	**RR-2 (n = 9)**	**SP (n = 10)**	**Test**
**Processing speed composite score**							
SDMT [61]	52.30 (12.34)	44.29 (9.63)	t = 2.60	48.31 (8.87)	40.00^*^ (9.24)	42.50 (9.71)	F = 3.47
			**p = 0.01**				**p = 0.02**
PASAT [52,53]	48.80 (10.44)	43.44 (11.06)	t = 1.74	48.54 (5.38)	34.67^*^ (12.49)	44.70 (11.31)	F = 4.65
			p = 0.089				**p = 0.006**
Forward Digit Span [62]	11.10 (1.92)	9.94 (1.83)	t = 2.19	10.38 (1.98)	10.00 (1.50)	9.30 (1.89)	F = 2.24
			**p = 0.033**				p = 0.10
Pegboard (Dominant/Non-dominant hand) [63]	27.40 (4.56)	33.95 (9.87)	t = 3.24	32.25 (9.29)	32.80 (5.48)	37.19^*^ (13.30)	F = 3.35
			**p = 0.002**				**p = 0.03**
**Memory composite score**							
SRT Immediate [64,65]	53.65 (6.85)	39.09 (10.96)	t = 5.32	42.08^*^ (11.62)	39.11^*^ (12.43)	35.20^*^ (8.15)	F = 14.31
			**p < 0.001**				**p < 0.001**
SRT CLTR [64,65]	36.85 (13.14)	18.00 (13.24)	t = 5.01	21.00^*^ (14.50)	19.11 (16.44)	13.10^*^ (6.49)	F = 13.85
			**p < 0.001**				**p < 0.001**
SRT Delayed [64,65]	8.10 (2.43)	5.53 (2.55)	t = 3.60	6.46 (2.50)	5.11^*^ (2.89)	4.70^*^ (2.11)	F = 5.67
			**p = 0.001**				**p = 0.005**
**Executive functions composite score**							
Tower of Hanoi, Time [60,66]	317.25 (139.78)	384.81 (132.47)	t = 1.74	417.67 (118.32)	390.67 (179.25)	340.11(95.56)	F = 1.61
			p = 0.088				p = 0.20
WCST 64-cards, correct sorts [67]	50.65 (3.88)	47.13 (7.71)	t = 2.18	48.92 (8.35)	44.11 (8.42)	47.50 (5.89)	F = 2.07
			**p = 0.034**				p = 0.14
WCST 64-cards, perseverative errors [67]	6.45 (2.11)	8.44 (5.65)	t = 1.46	7.85 (5.06)	8.44 (4.10)	6.90 (2.92)	F = 0.83
			p = 0.15				p = 0.48
Backward Digit Span [62]	7.65 (1.79)	6.22 (2.01)	t = 2.60	6.77 (1.79)	5.78 (2.82)	5.90^*^ (1.37)	F = 3.14
			**p = 0.012**				**p = 0.046**
FAS Total Correct [68]	46.50 (10.59)	36.69 (9.10)	t = 3.55	38.15 (10.37)	36.33^*^ (9.14)	35.10^*^ (7.84)	F = 4.28
			**p = 0.001**				**p = 0.009**
SRT Intrusions [64,65]	Md. 0.00 (Rg. 0–12)	Md. 1.00 (Rg. 0–6)	U = 230 p = 0.09	Md. 1.00 (Rg. 0–3)	Md. 0.00 (Rg. 0–4)	Md. 1 (Rg. 0–6)	χ^2^ = 5.26
							p = 1.54

*Abbreviations:* *CLTR* Continuous long-term retrieval; *FAS* Phonemic (letter) Fluency; *HCs* Healthy controls; *Md* median; *PASAT* Paced Auditory Serial Addition Test; *Rg* Range; *RR-1* Relapsing-remitting MS patients with EDSS (Expanded Disability Status Scale) scores 0–2.5; *RR-2* Relapsing-remitting MS patients with EDSS scores ≥ 3; *SP* Secondary progressive MS patients; *SDMT* Symbol Digit Modalities Test; *SRT* Selective Reminding Test; *WCST* Wisconsin Card Sorting Test, 64-card version.

^*^Significant difference to healthy controls (post-hoc Dunnett t-test or Bonferroni-corrected U-test).

Data are means (standard deviations) or medians (ranges).

### Game-of-Dice Task (GDT)

The computerized GDT assessed decision-making under explicit risk, and has been described previously in detail [16]. Briefly, the goal of the task is to maximise a virtual balance of $1000 in 18 play-rounds by betting on the outcome of a single dice throw. Betting choices include single, 2-, 3- and 4-number combination bets ranging in winning/losing probabilities and possible pay-outs (i.e., 1-number bets: 1:6 chance of winning and 5:6 chance of losing $1000; 4-number bets: 4:6 chance of winning and 2:6 chance of losing $100). Choices with a less than 50% chance of winning (1- and 2-number bets) are termed ‘high-risk’. Choices with at least 50% winning probability (3- and 4-number bets) are termed ‘low-risk’. Choice options and possible pay-outs are explicitly explained and are displayed throughout the task. Hence, the GDT assesses decision-making under explicit risk. The main outcome measure is the GDT net-score (low-risk minus high-risk choices; minimum = −18, maximum = +18, high scores indicate less risky choices). Another outcome measure is ‘strategy shifts’, which is the sum of alternations between high-risk and low-risk GDT choices (maximum = 17). Strategy shifts reflect errors in using feedback to develop and maintain a coherent choice strategy across the entire length of the GDT [30].

### MRI scans

Retrospective clinical MRI scans were available from the medical charts of 31 of the 32 MS patients and from the 20 non-MS control patients. Non-MS patients’ cranial MRIs were deemed unremarkable by their treating physicians. Given the nature of this recruitment strategy, scans were performed in different centres with a variety of protocols. Details on each participants’ scan protocol for the T2-weighted MRIs used for the ventricular measures are provided in Additional file 2. All scans were acquired on 1.5 Tesla MR scanners. In all but 4 cases (2 MS, 2 non-MS), a 5 mm slice thickness and 6.5 mm inter-slice gap was used. For the MS patients, average time between MRI and cognitive testing was 9.52 ± 12.88 months. The main purpose for including ventricular measures from the non-MS control patients was to compare ventricular widths on similar retrospectively sampled clinical MR scans against those of the MS patients. In past studies, linear ventricular width measures, especially third ventricle width (TVW) and intercaudate distance/ intercaudate ratio (ICD/ICR) have differentiated well between MS patients and healthy controls [2,12,38,39]. Thus, our approach to include a non-MS patient group as a reference can be considered conservative. In the MS patients only, we correlated ventricular width measures with EDSS and disease duration to test whether these measures reflected MS severity/progression in our sample, similar to approaches in past studies [39-41].

### Ventricular width measures

Linear measurements of ventricular width were performed on the MR images using a digital ruler in ClearCanvas Workstation 2.0. (https://www.clearcanvas.ca). Measures were made on the most caudal axial T2-weighted slice where the frontal horns appeared to reach maximal width [9] (see Additional file 3 for an illustrated example). Measures included: 1) Frontal Horn Width (FHW): Maximal distance between the lateral borders of the frontal horns of the lateral ventricles; 2) Intercaudate Distance (ICD): Minimum distance between the medial borders of the head of the caudate nuclei; 3) Third Ventricle Width (TVW): Maximum distance between the lateral borders of the middle of the third ventricle where the ventricles’ borders are most parallel; 4) Frontal Horn Ratio (FHR): FHW divided by the Transverse Width (TW): Minimum distance separating the inner tables of the skull at the level of the caudate nuclei; 5) Intercaudate Ratio (ICR): ICD divided by the TW; 6) Third Ventricle Ratio (TVR): ICD divided by the TW. Thus, we recorded three absolute measures (FHW, ICD, TVW) and three ratios (FHR, ICR, TVR) correcting the three ventricular width measures for brain width. The three ventricular width measures as well as the transverse width were recorded three times (A.D.R.) yielding excellent intra-rater reliability (intra-class coefficients: FHW = 0.992; ICD = 0.995; TVW = 0.997; TW = 0.989). An average of these three measures was used in the analyses and to derive the ratio measures. A second rater repeated all measures, yielding high inter-rater reliability (FHW = 0.876; ICD = 0.983; TVW = 0.968; TW = 0.987).

The use of different imaging facilities, scanner models, and imaging protocols in a retrospective sampling approach such as here could conceivably lead to systematic biases. Seven different scan sites were included, with 38 of the 51 scans (24 MS patients,14 non-MS control patients) from site 1. Sites 2–7 had between 1 and 3 scans per site with a total of 13 scans from sites 2–7 (7 MS patients, 5 non-MS control patients). Furthermore, scans were from six different MR scanners. More than half of the scans (n = 29) were acquired with a Siemens Avanto scanner model (21 MS patients, 8 non-MS control patients), with substantially fewer scans from the additional five scanners (details see Additional file 2). The small total number of scans and imbalanced frequencies of individual scans per site/scanner precluded a direct comparison of the ventricular width measures between each individual site/scanner. In order to allow some comparison and quality control nevertheless, we dichotomised ‘scan site’ and ‘scanner model’, and tested differences in the ventricular width measures between sites (site 1 versus sites 2–7), and between scanners (Siemens Avanto vs. other scanner models). Briefly, none of the comparisons yielded significant differences in any of the six ventricular width measures between scan sites or scanner models (see Additional file 4 for details). Thus, despite the limitations of this retrospective MR sampling method, we did not observe systematic biases in the ventricular measures across scan sites or scanner models.

### Statistical analyses

Statistical standard procedures were carried out with SPSS 22.0 for Windows (IBM SPSS Statistics). Data were tested for normality with Kolmogorov-Smirnoff test. Non-normality was observed in the following variables: Intrusions in the Symbol Digit Modalities Test, third ventricle width, third ventricle ratio, GDT strategy shifts. The EDSS score was marginally normal (p = 0.052). Comparisons between two groups (MS patients versus healthy controls) were carried out with t-tests or with non-parametric U-tests, if indicated. Patient subgroups (RR-1, RR-2, and SP) were contrasted to controls by analysis of variance and post-hoc Dunnett t-tests, correcting for multiple comparisons. As non-parametric equivalent, the Kruskall Wallis test and post-hoc Bonferroni-corrected U-tests against controls were used, if indicated. Moderated regression analyses were carried out to control for demographic differences between patient subgroups potentially influencing decision-making performance. Simple correlations between variables were assessed with Pearson or Spearman rank correlations, respectively. Partial correlations were Pearson correlations, with preceding log-transformation of non-normal variables. To limit the total number of correlations, only ventricular ratios (not absolute ventricular width measures) were correlated with disease parameters and cognitive variables. However, even despite these restrictions and the use of cognitive composite scores instead of individual tests, the total number of correlations carried out here could inflate type-1 error. To retain some sensitivity in light of the small sample size, we report both, the uncorrected correlation results and those adjusted by false-discovery rate accounting for the number of correlations [42]. A structural equation model to test mediation effects was carried out using the MPlus modelling framework [43]. Before testing the model, the fits of the latent dimensions underlying ventricular widening were tested using confirmatory factor analysis (CFA), also in MPlus. For both, mediation analysis and CFA, maximum likelihood parameter estimation was applied. Standard criteria were used for the evaluation of model fits [44,45]. These included: Standardized root mean square residual (SRMR; values below .08 indicate a good fit with the data), comparative fit indices (Bentler’s comparative fit index [CFI] and Tucker-Lewis index [TLI]; values above .90 indicate a good fit, values above .95 an excellent fit), and root mean square error of approximation (RMSEA; “test of close fit”; a value below .08 with a significance value below .05 indicates an acceptable fit).

## Results

### Neuropsychological performance and psychosocial variables

Results on the individual tests contained within the cognitive composite scores are shown in Table 3.

MS patients showed significantly lower performance than healthy controls on the composite scores for processing speed, memory, and executive functions, and consequently also in global cognitive functioning (Table 2).

Comparing individual MS patient subgroups against healthy controls, the RR-1 group underperformed in memory and global cognitive functions, the RR-2 group scored below healthy controls in all domains, and the SP group was impaired in all domains except executive functions. Compared to published cut-off scores, MS patients reported severe fatigue, but no elevated levels of anxiety or depression, and only mild dysexecutive symptoms (see Additional file 1).

### Game-of-Dice Task (GDT)

**Figure 1:**
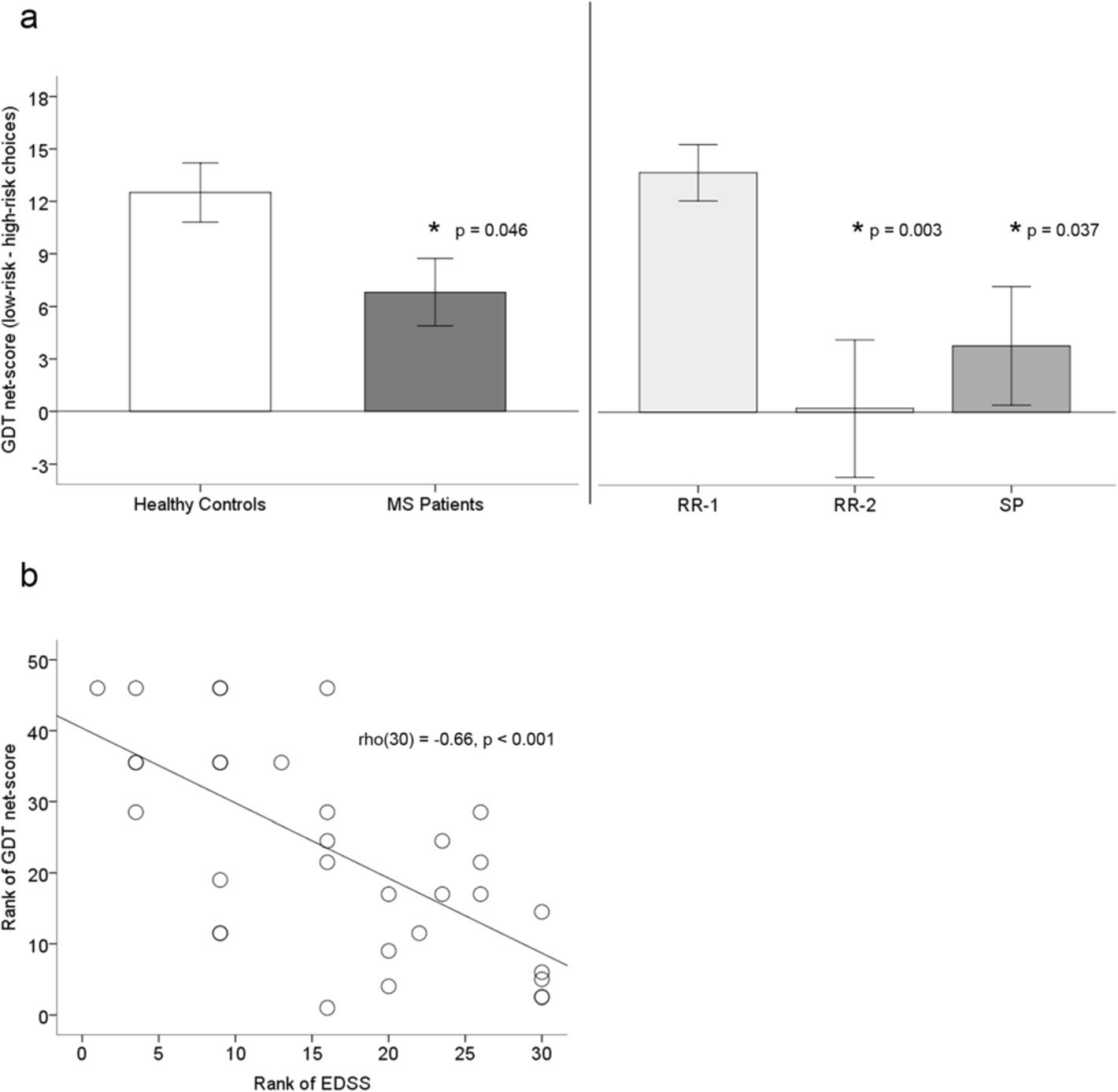
GDT performance in MS patients and healthy controls. **(a)** Mean GDT net-score (low-risk minues high-risk choices) in healthy controls compared to all MS patients and compared to MS patient subgroups. **(b)** Spearman rank-correlation between MS patients’ EDSS score and GDT net-score. EDSS: Expanded Disability Status Scale; GDT: Game-of-Dice Task; RR-1: Relapsing-remitting MS patients with EDSS scores < 3; RR-2: Relapsing-remitting MS patients with EDSS scores ≥ 3; SP: Secondary progressive MS patients. Error bars are standard errors of the mean.

Among all MS patients, the mean GDT net-score was significantly lower than that of the healthy controls (Table 2; Figure 1(a)).

However, comparing the different MS subgroups with healthy controls showed that GDT net-scores were significantly reduced only in the RR-2 and SP groups. This finding was echoed by negative correlations between GDT net-score and EDSS (r[27] = −0.59, p < 0.001; Spearman rho[27] = −0.66, p < 0.001) (Figure 1 (b)), as well as GDT net-score and MSSS (r[27] = −0.42, p = 0.016). Individual MS subgroups also differed from healthy controls in the number of GDT strategy shifts, but only the SP-MS group demonstrated significantly increased shifting (Table 2). Thus, overall, decision-making in the GDT was reduced only in the RR-2 and SP groups.

Of note, controlling for the marginally lower education and premorbid IQ in the RR-2 and SP subgroups (see Table 1), did not change these results. In detail, we conducted three moderated multiple regression analyses [46] predicting GDT net-score by years of education and premorbid IQ (step 1), subgroup membership (step 2: membership in the subgroups RR-1, RR-2, or SP were dummy-coded with healthy controls as a reference group) and their 2-way interactions (step 3). Years of education and premorbid IQ by themselves did not predict GDT net-score well (R^2^ = 0.007, F [2,47] = 0.17, p = 0.85). Including subgroup (step 2) rendered a significant model (R^2^ = 0.34; F_change_ [3,45] = 7.70, p < 0.001; F[5,45] = 4.72, p = 0.001), but including the interaction terms in step 3 (R^2^ = 0.46, F[11,37] = 3.09, p = 0.004) did not substantially increase the prediction of GDT variance over step 2 (F_change_ [6,37] = 1.49, p = 0.21). The regression weights in step 2 showed that RR-2 subgroup membership (b = −14.85, standard error (SE)_b_ = 3.76, β = −0.57, t = −3.95, p < 0.001) and SP subgroup membership (b = −11.16, SE_b_ = 3.62, β = −0.44, t = −3.09, p = 0.003) both predicted lower GDT net-scores compared to controls, whereas membership in the RR-1 subgroup did not (b = 0.97, SE_b_ = 3.08, β = 0.04, t = −0.32, p = 0.75). Thus, the GDT net-score reductions in the RR-2 and SP-subgroups were not substantially influenced by demographic characteristics of these patients.

### Ventricular width

Ventricular width measures have been applied to MS in the past to approximate atrophic brain changes [2,6,7,9,10,12,13]. These indices have been correlated with MS disease progression [40,48] as well as with neuropsychological impairments [2,6,7]. Compared to the 20 gender- and age-matched non-MS control patients, MS patients exhibited significantly larger ICD/ICR and TVW/TVR (Table 4). The ICR measure further differentiated patient subgroups (F[3,46] = 3.67, p = 0.019), with larger ICR in the RR-2 (p = 0.046) and SP groups (p = 0.019) compared to the non-MS control patients (post-hoc Dunnett-t tests). Of note, the TVW measured 2.3 mm (median) or 3.3 mm (mean) in our MS group, similar to previously reported mean TVW in MS cohorts ranging between 3.0 mm [11], 3.12 mm [9], 3.58 (RR-MS), and 5.04 (SP-MS) [2]. The FHW (32.26 mm) and ICD (12.2 mm) were likewise similar to previously reported cohorts (FHW: 33.33 mm, ICD: 12.12 [9]) of MS patients. Controlling for age, gender, scan site, and scanner model, EDSS was positively correlated with TVR (Table 5).

**Table 4:** **Ventricular width/ratio measures in MS and non-MS control patients**

	**Non-MS patients (n = 20)**	**MS patients (n = 31)**	**Test**
**Female, n (%)**	12 (60.00%)	23 (74.19%)	χ^2^ = 1.14
			p = 0.29
**Age, years**	47.8 (12.08)	50.5 (9.43)	t = 0.88
			p = 0.38
**FHW, cm**	3.18 (0.38)	3.26 (0.28)	t = 0.85
			p = 0.40
**ICD, cm**	1.04 (0.24)	1.22 (0.27)	t = 2.48
			**p = 0.02**
**TVW, cm**	0.17 (0.11 - 0.7) ^a^	0.23 (0.1 - 1.0) ^a^	U = 190
			**p = 0.02**
**FHR, cm**	0.28 (0.02)	0.29 (0.03)	t = 1.23
			p = 0.22
**ICR, cm**	0.09 (0.02)	0.11 (0.02)	t = 2.83
			**p = 0.007**
**TVR, cm**	0.02 (0.01 - 0.08) ^a^	0.01 (0.01 - 0.06) ^a^	U = 188
			**p = 0.02**

*Abbreviations:* *FHW* Frontal horn width; *ICD* Intercaudate distance; *TVW* Third ventricle width; *FHR* Frontal horn ratio; *ICR* Intercaudate ratio; *TVR* Third ventricle ratio.

^a^ Medians (ranges).

Data are means (standard deviations) or medians (ranges).

**Table 5:** **Partial correlations between ventricular ratio measures, disease parameters and cognition in MS patients** ^a^

	**FHR**	**ICR**	**TVR (log)**
**Disease duration**	0.27	0.31	0.32
	p = 0.17	p = 0.11	p = 0.11
**EDSS**	0.13	0.34	0.38
	p = 0.52	p = 0.09	**p = 0.049**
**Processing speed**	−0.15	−0.57	−0.53
	p = 0.46	**p = 0.002** ^**b**^	**p = 0.005** ^**b**^
**Memory**	−0.01	0.003	−0.12
	p = 0.98	p = 0.99	p = 0.57
**Executive functions**	0.13	−0.01	0.04
	p = 0.52	p = 0.97	p = 0.83
**Global cognitive function**	0.02	−0.21	−0.23
	p = 0.92	p = 0.30	p = 0.26
**GDT net-score**	0.03	−0.42	−0.52
	p = 0.88	**p = 0.03**	**p = 0.007** ^**b**^
**GDT strategy shifts (log)**	−0.34	−0.02	0.01
	p = 0.09	p = 0.93	p = 0.98

*Abbreviations:* *EDSS* Expanded Disability Status Scale; *FHR* Frontal horn ratio (cm); *GDT* Game-of-Dice Task; *ICR* Intercaudate ratio (cm); *TVR* Third ventricle ratio (cm).

^a^ Partial correlations with disease duration and EDSS controlled for age, gender, scan site, and scanner model (df = 25). Partial correlations with cognitive composite scores and GDT-scores additionally controlled for time between MR scan and test date (df = 24).

^b^ p < 0.05, corrected by False-Discovery Rate [42].

### Associations between GDT, cognitive performance and brain atrophy

Correlations were not performed within MS patient subgroups due to the small subgroup sample sizes. Neither GDT net-score nor strategy shifts were significantly correlated with cognitive performance in healthy controls, likely due to ceiling/floor effects. In the MS patients, the GDT net-score was positively correlated with the processing speed composite score (r[27] = 0.41, p = 0.019; although not significant after False-Discovery Rate correction [42]). The GDT net-score was not correlated with any of the other cognitive composite scores (all p’s > 0.1). Within the processing speed composite, all individual tests correlated with the GDT net-score in the expected direction; the SDMT showed the strongest individual correlation (r[27] = 0.37, p = 0.043, not significant after correction [42]). Even though the composite score of executive functions did not correlate with GDT net-score, we had strong *apriori* expectations to observe such relationships based on prior studies [16,29,30]. Thus, we also examined relationships between the GDT net-score and individual executive function tests. We observed, in MS patients only, that the number of WCST correct sorts was correlated with the GDT net-score (r[27] = 0.38, p = 0.032, not significant after correction [42]). None of the self-report psychosocial or symptom questionnaires correlated with the GDT net-score in MS patients. Controlling for age, gender, scan site, scanner model, and time between MR scan and test date, the ventricular ratio measures ICR and TVR were negatively correlated with MS patients’ GDT net-score as well as with processing speed (Table 5). Ventricular width was unrelated to the other composite scores and to GDT strategy shifts.

**Figure 2:**
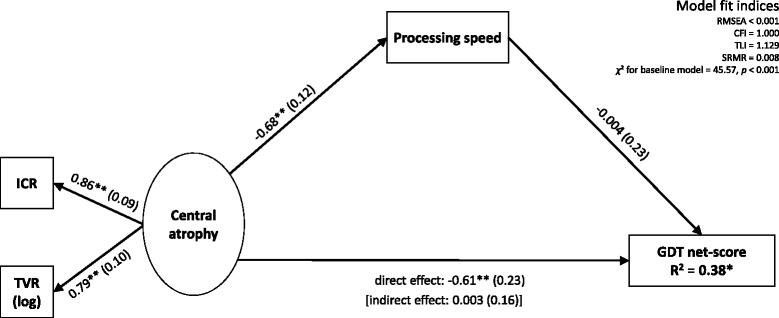
Mediation model of central atrophy (latent variable) predicting processing speed (observed) and GDT net-score (observed) [28]. Model fits were adequate according to RMSEA (root mean square approximation), CFI (Bentler's Comparative Fit Index), TLI (Tucker-Lewis Index), SRMR (Standardized Root Mean Square Residual), and χ^2^. Coefficients (standard errors) are standardised. GDT: Game-of-Dice Task; ICR: Intercaudate ratio; TVR: Third ventricle ratio. ^*^ p < 0.05; ^**^ p < 0.01.

To recapitulate, EDSS-based disability was only marginally related to the TVR (Table 5), but differentiated MS-patients well on all cognitive composite scores, the GDT net-score and the GDT stategy shift score (Table 2). The ventricular ratios ICR and TVR in turn were more selectively related to the processing speed composite and to the GDT net-score (Table 5). To clarify whether the GDT net-score was fully or partly mediated by processing speed and to rule out a possible redundancy between the GDT and tests of processing speed, we conducted a mediation model on the GDT net-score using MPlus [43]. Predictors were ICR and the log-transformed TVR (modelled together as latent variable “central atrophy”); the processing speed composite score was used as a mediator. We did not include any additional covariates or mediators in this model (i.e., age, gender, scan site, scanner model, or delay between test date and scan date). These variables did not show significant bivariate correlations with GDT net-score and with ICR/TVR; therefore they did not fulfil the necessary criteria for mediation [43]. Standard model fits indicated a good representation of our data by this model (see Figure 2).

In total, 38% of the variance in MS patients’ GDT net-score was explained by central atrophy and processing speed. However, whereas the direct effect of central atrophy on GDT net-score was highly significant (p = 0.008), the indirect effect via processing speed was decidedly not significant (p = 0.99). Thus, GDT performance was not mediated by processing speed. Instead, central atrophy independently predicted both slowed processing speed as well as disadvantageous decisions in the GDT in the MS patients.

## Discussion

Our goals were to test whether the GDT as a non-speeded measure of decision-making under explicit risk is impaired in MS, and to evaluate whether GDT performance is linked to increasing disability and to ventricular width. We further tested whether we could observe correlations between GDT performance, executive functions, and processing speed. Finally, we assessed whether potential associations between GDT performance and ventricular width were mediated by other cognitive functions.

We observed reductions in the GDT in MS patients, but limited to patients with more severe disability. Patients’ decision-making performance was correlated with enlarged intercaudate ratio and third ventricle ratio. We also observed a relationship between processing speed and GDT performance in the MS-patients, but no strong links to executive functions. The relationship between processing speed and GDT performance was fully explained by intercaudate/ third ventricle ratios, pointing to a role of central atrophy in decision-making regardless of processing speed deficits in MS.

In addition to white matter lesions, demyelination and/or neuronal loss in grey matter has been highly predictive of cognitive deficits in MS [2,5-7]. Simple linear ventricular width measures from routine clinical MRI scans offer reasonably stable estimates of whole-brain or regional atrophy [2,8,9,12,13,40,49]. For example, Turner et al. (2001) [12] reported a significant correlation of r = 0.81 between the third ventricle width assessed on 2-D MRI and the 3-D volume of the third ventricle in 20 relapsing-remitting (RR) and 20 secondary progressive (SP) MS patients. Sharma et al. [13] reported similarly high correlations between whole-brain/parenchymal fraction assessed via 3-D MRIs and linear ventricular width measures (bicaudate ratio: r = −0.74, third ventricle: r = −0.81) in 52 MS patients (43 RR-MS and 9 SP MS). Bermel et al. [8] found 3-D brain/parenchymal fraction inversely correlated with third ventricular width (r = −0.79, p < 0.001) in 78 MS patients. Significant correlations were also reported by Butzkueven et al. [9]: Intercaudate ratio (r = −0.45) and third ventricular ratio (r = −0.65) were inversely correlated with volumetric brain/parenchymal fraction in 35 MS patients. Indeed, Cifelli et al. [49] reported a negative correlation of r = −0.59 between 2-D MRI-based third ventricle width and thalamic neuron count in a post-mortem study of 14 secondary progressive MS patients. Thus, 2-D ventricular width measures, especially third ventricle width, have shown moderate to high correlations with whole-brain or regional atrophy in MS. Correlations between linear ventricular width measures and clinical variables in MS (disability, disease progression, MS subtype) as well as cognitive functions have been well-studied [2,6,7,10,13-15,40,41]. The TVW/TVR and ICR have been particularly predictive of MS-related cognitive impairment [2,7,10], echoing the present findings. Of note, the present study does not claim to examine the absolute extent of ventricular enlargement in the MS sample, which is likely underestimated due to our use of neurological controls for validation of the measures.

Compared to the RR-1 and the SP patient subgroups, the RR-2 subgroup was most impaired in the GDT and across all of the composite scores. Among the possible reasons for this unexpected finding may be characteristics of the subgroups: Some of the RR-1 patients may have had a ‘benign’ subtype of MS. In turn, the SP patients could be considered as being in a stable state of disease progression without experiencing any remissions. In this regard, a previous decision-making under ambiguity study [21] found that RRMS patients without recent (>15 months) MS relapses had higher executive function scores than patients with recent relapses. Although we did not measure relapses in this study, it is possible that the RR-2 patients were at a stage of transitioning into an SP subtype. The conversion from an RR to an SP subtype is marked by an increase in cognitive impairment [47,50], accompanied by accelerated degeneration of the cerebral grey matter [51]. One could speculate then that the apparent cognitive dysfunctions as well as decision-making impairment in the RR-2 group may reflect a currently less stable disease state than in both RR-1 and SP patients here. However, in light of the very small group sizes, this remains speculative.

To our knowledge, decision-making under explicit risk in MS-patients has been assessed in four studies, two also using the GDT [23,25] and two using the Cambridge Gambling Task (CGT [27,28]). The CGT is a *timed* task and, although MS patients were not substantially impaired in the CGT, their decision deliberation times were slowed in Simioni et al. [28]. Perhaps not surprisingly, patients’ slowed CGT decisions were related to processing speed (in the Paced Auditory Serial Addition Task [52,53]). In Muhlert et al. [27], decision deliberation time was also the most sensitive CGT metric to differentiate MS patients from healthy controls, although here the correlations were more general involving processing speed, memory, and executive functions. The MS patients in Farez et al. [23] were impaired in the untimed GDT, again correlated with processing speed. These findings together with the current GDT findings suggest that slowed processing speed may represent a domain-general deficit in MS, underlying deficits in untimed tasks [54], and extending to decision-making in the GDT.

However, GDT performance has not been related to processing speed in non-MS samples [16,29], and since the GDT is untimed, it seems unlikely that slowed processing speed could directly affect GDT performance. Furthermore, comparing the three MS cohorts studied with the GDT so far (our study, [23], [25]), it appears that potential MS-related GDT-deficits should be viewed in light of the disease course and cognitive deficits. Intact GDT performance in our RR-1 patients and in the RR-MS patients of Cogo et al. [25] contrasted GDT impairment reported by Farez et al. [23]. Cogo et al’s patients were cognitively intact, our RR-1 group was only impaired in verbal memory, and Farez et al’s sample was impaired in processing speed, visual memory, and verbal fluency. The other core variable differentiating the three samples was disease duration, ranging from 7.9 months in Farez et al., to 3.3 years in Cogo et al., and reaching more than 17 years in our RR-1 group. Although speculative, it is possible that Farez et al. [23] included individuals with a more aggressive disease course, relative to our RR-1 group and to Cogo et al. [25]’s patients. Farez et al.’s finding of a link between GDT performance and processing speed (as well as visual memory) could then perhaps be understood as accompanying a more aggressive disease course.

Our and other findings [18,27] suggest that similar to other cognitive functions, decision-making abilities in MS can deteriorate with disease progression or severity of functional disability. EDSS-based disability was more generally related to all cognitive composite scores here, including also GDT performance (Table 2). The ventricular measures (ICR, TVR) were more selectively correlated only with the processing speed composite score and with the GDT net-score, and only the TVR was related to EDSS (Table 5). Relationships between brain atrophy, physical disability and cognition in MS are complex. EDSS-based disability covaries with cognitive status, and in turn cognitive functions can predict also physical health-related quality of life [55,56]. In direct comparison, MS-related brain changes, especially grey matter loss, have been more predictive of cognitive impairment in MS than physical disability measured by the EDSS [57,58]. However, the ventricular width measures here are a rather coarse estimate of brain atrophic changes. It therefore remains possible that more direct assessment of actual loss of brain tissue/volume would have been more sensitive to the additional cognitive deficits we found covarying with the EDSS score here.

Our mediation results speak more directly to the possibility that decision-making abilities in MS can deteriorate with disease progression. As such, MS progression indicated by central atrophy, independently predicted both processing speed deficits and decision-making deficits in our sample. In addition, the relationship between the two cognitive functions was eliminated when accounting for atrophy. The question remains then what aspects of the GDT, besides slowing in processing speed, may underlie the link with the central atrophy measures we observed here. We found some evidence for a correlation between specific aspects of executive functions (correct sorts in the Wisconsin Card Sorting Test) and decision-making. Several studies have demonstrated that the GDT net-score correlates with performance in card sorting tests [16,29,30,59]. These tasks assess the set-shifting component of executive functions [60]. If set-shifting is a crucial executive function involved in the GDT, using a composite executive function score might have eclipsed its overall relationship with GDT. Furthermore, the GDT also involves reward-processing from choice feedback. We did not test MS patients’ ability to process emotional feedback in the GDT, since the GDT is not designed to obtain such a measure precisely. Nevertheless, it is conceivable that ventricular enlargement would also covary with reward-related aspects of the GDT. Disturbances in communication between striatal and prefrontal brain regions, especially dorsolateral prefrontal cortex, underlie deficits in decision-making under explicit risk [26,29]. Indeed, in Muhlert et al. [27] grey matter atrophy in the caudate and grey matter changes assessed via diffusion MRI (diffusion orientational complexity) in middle and medial prefrontal regions covaried with CGT-based decision-making speed in MS patients. Relationships between structural brain changes and other cognitive functions (especially processing speed) covarying with the CGT were not assessed in Muhlert et al. Therefore, it would be interesting to test whether similar networks of brain regions are involved in decision-making without a time restriction, such as in the GDT. In general, emotional/reward-related elements of decision-making [18] still play a role even in decisions under explicit risk. That is, in addition to the cognitive/executive ability for making advantageous choices based on the use of information about the winning/losing odds, each trial in the GDT is also followed by reward or punishment feedback. The contribution of MS-related brain changes to the proper use of feedback in decision situations should be explored further, using additional tasks like the IGT, more contemporary neuroimaging techniques and larger cohorts.

### Strengths and limitations

The strengths of this study include the use of a decision-making task that does not rely on speeded responses, given that general slowing in speeded functions is prominent in MS [3]. Furthermore, using a mediation model we could uncover that processing speed deficits, although showing a bivariate correlation with decision-making, were rendered insignificant when taking central atrophic brain changes into account. Understanding ventricular enlargement measures as indices for disease severity/progression, both processing speed deficits and decision-making deficits may develop independently from each other in the course of MS. Thus, our results complement and extend the existing small body of literature on decision-making in MS by suggesting to explore contributions of MS-related brain changes to aspects of decision-making that are separable from processing speed deficits.

Among the limitations, it should be noted that the results of this study are preliminary based on the small sample size, especially with regard to our analyses in subgroup samples. We intended the splitting of our RR-MS subgroup to reflect the variability of cognitive impairment in general, and decision-making deficits in particular across the range of disability in MS. Since the correlation between GDT performance and EDSS scores achieves the same outcome, one could alternatively avoid splitting the RR subgroup and treat EDSS (or MSSS) solely parametrically, possible only in larger samples. A more targeted inclusion of ‘benign’ MS patients, better-matched in demographic background, would also be helpful to clarify the exact characteristics of MS patients with intact GDT performance. Evidently, acquiring standardized, high-resolution MR allowing regional and whole-brain volume assessment would be more precise than relying on retrospective sampling of rather heterogeneous 2-D MRIs. Additional MS-related brain changes (e.g., lesion load) or whole-brain atrophy measures (e.g., brain-parenchymal fraction) would also be of interest to explore in addition to the ventricular width measures. Such could address questions surrounding different types and regional specificity (if any) of MS-related brain pathologies to decision-making in the GDT.

Furthermore, the MS patients were currently taking a number of medications with potential influences on cognitive functions. The sample size precluded any further analyses based on current medication status, but in larger cohorts, medication status should be considered as a potentially important covariate. However, we would like to point out that despite the current medications for symptom management and mood, self-report questionnaires with regard to psychological problems did not point to major psychiatric comorbidities in our group (see Additional file 1). Finally, patients were not currently experiencing relapses and were not treated with corticosteroids. Historical information about past relapse rates and preceding treatment with corticosteroids was unfortunately not available at the time of ascertainment of participants and such has to be taken as a caveat.

## Conclusions

Decision-making under explicit risk, as measured with the GDT, was impaired in MS patients as a function of disability severity. GDT performance was correlated with processing speed in MS patients, even though the GDT is an untimed decision-making task. However, both slowed processing speed and GDT deficits were independently explained by the degree of central atrophy. These results imply that MS-related atrophic brain changes may contribute to decision-making deficits in the GDT irrespective of their involvement in general processing speed deficits. Individuals with MS are confronted with complex health-related decisions concerning diagnostic and treatment interventions that require proper handling such that a compromised ability to oversee consequences of one’s decisions could have wide-ranging effects. Our results may imply that providing additional support (e.g., additional time to make a decision) in those types of decision situations, especially for individuals in more advanced stages of the disease, could be beneficial.

## Additional files

Additional file 1:
**Psychosocial and symptom questionnaire scores of MS patients compared to published norms/cut-off scores.**
Additional file 2:
**Details of the T2-weighted axial 2-D MRIs used for the ventricular width measures.**
Additional file 3:
**Example 2-D linear measurements on a T2-weighted axial magnetic resonance image.** A: Frontal Horn Width (FHW); B: Intercaudate Distance (ICD); C: Transverse Width (TW; used as denominator for the Frontal Horn Ratio (FHR = FHW/TW), Intercaudate Ratio (ICR = ICR/TW) and Third Ventricle Ratio (TVR = TVW/TW); D: Third Ventricle Width (TVW). Additional file 4:
**Comparisons of ventricular width measures by scan site and scanner model (31 MS patients and 20 non-MS control patients).** Data are means (standard deviations) or medians (ranges).

## Abbreviations

CFA: Confirmatory factor analysis
CFI: Bentler’s comparative fit index
CGT: Cambridge Gambling Task
EDSS: Expanded Disability Status Scale
FHR: Frontal horn ratio
FHW: Frontal horn width
GDT: Game-of-Dice Task
ICD: Intercaudate distance
ICR: intercaudate ratio
IGT: Iowa Gambling Task
MRI: Magnetic resonance imaging
MS: Multiple sclerosis
MSSS: Multiple Sclerosis Severity Score
RMSEA: Root mean square error of approximation
RR: Relapsing-remitting
SP: Secondary progressive
SRMR: Standardized root mean square residual
TLI: Tucker-Lewis index
TVR: Third ventricle ratio
TVW: Third ventricle width
TW: Transverse width
