# Supplementary material for: Decision-making under explicit risk is impaired in multiple sclerosis: relationships with ventricular width and disease disability
Source: BMC Neurol. 2015 Apr 23;15:61. doi: 10.1186/s12883-015-0318-0 (PMC4428249; doi:10.1186/s12883-015-0318-0)
Supplement: Additional file 3: — Example 2-D linear measurements on a T2-weighted axial magnetic resonance image. A: Frontal Horn Width (FHW); B: Intercaudate Distance (ICD); C: Transverse Width (TW; used as denominator for the Frontal Horn Ratio (FHR = FHW/TW), Intercaudate Ratio (ICR = ICR/TW) and Third Ventricle Ratio (TVR = TVW/TW); D: Third Ventricle Width (TVW). [file 12883_2015_318_MOESM3_ESM.pdf]

Additional file 3

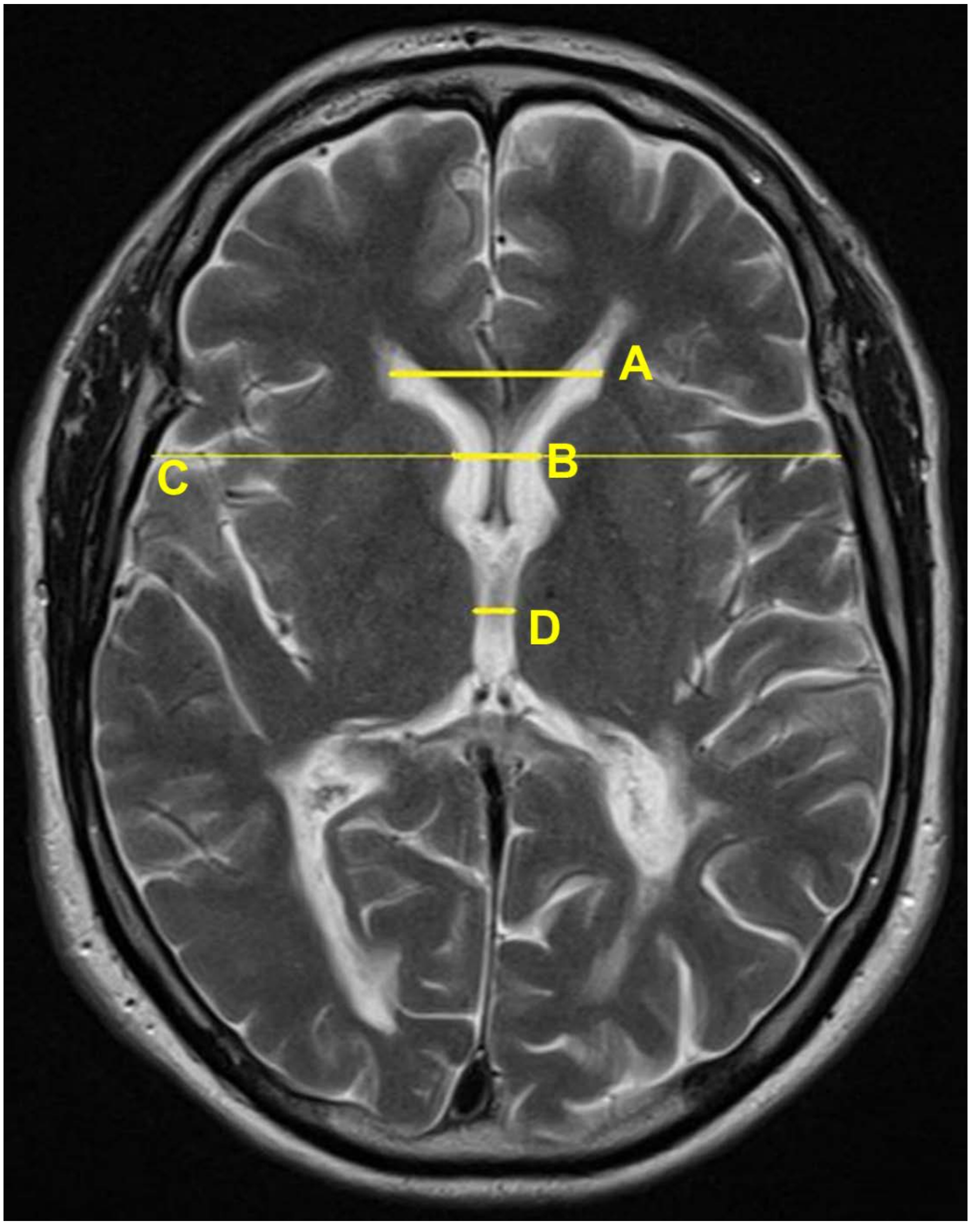

Figure caption: Example 2-D linear measurements on a T2-weighted axial magnetic resonance image. **A**: Frontal Horn Width (FHW); **B**: Intercaudate Distance (ICD); **C**: Transverse Width (TW; used as denominator for the Frontal Horn Ratio (FHR = FHW/TW), Intercaudate Ratio (ICR = ICR/TW) and Third Ventricle Ratio (TVR = TVW/TW); **D**: Third Ventricle Width (TVW).
